# Analysis of the hikikomori phenomenon – an international infodemiology study of Twitter data in Portuguese

**DOI:** 10.1186/s12889-023-17617-0

**Published:** 2024-02-19

**Authors:** Francisca Correia Lopes, Mariana Pinto da Costa, Cesar I Fernandez-Lazaro, Francisco J Lara-Abelenda, Victor Pereira-Sanchez, Alan R Teo, Miguel Angel Alvarez-Mon

**Affiliations:** 1 Institute of Biomedical Sciences Abel Salazar, University of Porto, Porto, Portugal; 2 Institute of Psychiatry Psychology & Neuroscience, King´s College London, London, UK; 3 Department of Preventive Medicine and Public Health, School of Medicine, University of Navarra, IdiSNA, Navarra Institute for Health Research, Pamplona, Spain; 4 Department of Signal Theory and Communications and Telematic Systems and Computing, School of Telecommunications Engineering, Rey Juan Carlos University, 28942 Madrid, Spain; 5 Child Mind Institute, New York, NY USA; 6 Health Services Research & Development Center to Improve Veteran Involvement in Care, VA Portland Health Care System, Portland, OR USA; 7 Department of Psychiatry, Oregon Health & Science University, Portland, OR USA; 8 Department of Medicine and Medical Specialties, Faculty of Medicine and Health Sciences, University of Alcala, Alcala de Henares, Spain; 9 Department of Psychiatry and Mental Health, University Hospital Infanta Leonor, Madrid, Spain; 10 Ramón y Cajal Institute of Sanitary Research (IRYCIS), Madrid, Spain

**Keywords:** Hikikomori, Loneliness, Social isolation, Mental health, Internet, Social media, Social withdrawal, Internet addiction

## Abstract

**Background:**

Hikikomori refers to the extreme isolation of individuals in their own homes, lasting at least six months. In recent years social isolation has become an important clinical, social, and public health problem, with increased awareness of hikikomori around the globe. Portuguese is one of the six most spoken languages in the world, but no studies have analysed the content regarding this phenomenon expressed in Portuguese.

**Objective:**

To explore the hikikomori phenomenon on Twitter in Portuguese, utilising a mixed-methods approach encompassing content analysis, emotional analysis, and correlation analysis.

**Methods:**

A mixed methods analysis of all publicly available tweets in the Portuguese language using a specific keyword (hikikomori) between 1st January 2008 and 19th October 2022. The content analysis involved categorising tweets based on tone, content, and user types, while correlation analysis was used to investigate user engagement and geographical distribution. Statistical analysis and artificial intelligence were employed to classify and interpret the tweet data.

**Results:**

Among the total of 13,915 tweets generated, in terms of tone 10,731 were classified as “negative”, and 3184 as “positive”. Regarding content, “curiosities” was the most posted, as well as the most retweeted and liked topic. Worldwide, most of the hikikomori related tweets in Portuguese were posted in Europe, while “individuals with hikikomori” were the users most active posting. Regarding emotion analysis, the majority of tweets were “neutral”.

**Conclusions:**

These findings show the global prevalence of the discourse on hikikomori phenomenon among Portuguese speakers. It also indicates an increase in the number of tweets on this topic in certain continents over the years. These findings can contribute to developing specific interventions, support networks, and awareness-raising campaigns for affected individuals.

## Background

Hikikomori is a Japanese concept that emerged in the last decades of the twentieth century and refers to extreme withdrawal by some people in their own homes, lasting at least six months [1]. This isolation usually begins in adolescence or early adulthood and affects a higher proportion of males [2]. Hikikomori is characterised by a lack of participation in education, work, and other daily life activities, resulting in profound suffering or functional impairment [3–5]. Hikikomori impacts not only individuals who suffered from this condition, but also their families who desperately try to help them with no success [6, 7].

Whilst the internet is increasingly present in our lives, and therefore, people are more digitally connected, this may sometimes lead to isolation and increased loneliness [8]. There may be different situations why people isolate themselves on the internet, from online gaming to social networking, as well as family problems and difficulties of inclusion in the school of young people [9, 10]. The COVID-19 pandemic has also led to an increased awareness of social isolation. Studies in different countries, such as Brazil, Portugal, England, China, Iran, Malaysia, Pakistan, Philippines, Thailand, and Vietnam, showed that the pandemic affected mental health, leading to anxiety, depression, and stress, which resulted in social isolation [11–14]. The pandemic brought many social changes, from mandatory physical distancing to quarantines, which may have reinforced hikikomori-prone behaviour in people with mental health conditions [15, 16].

Infodemiology is the science that includes the collection, analysis and interpretation of health-related information in electronic media (internet and other digital sources) with the aim of informing public health and public policy [17, 18]. The internet is essential for sharing opinions, knowledge, and problems, and online social networks allow new forms of (online) communication and information sharing [19]. Twitter stands out as a prominent social network, valued for its short messages with a limit of 280 characters (currently up to 25,000 for subscribers of the premium version), with the possibility of anonymity [19–22]. Users can also repost content from other users (retweet), which makes them reach a larger population, sometimes marking their tweets with hashtags to identify a theme and allow other users to see related tweets [23, 24]. Recent research has embraced the analysis of tweets to explore, identify, and reach individuals with particular characteristics of interest to seek their perspectives, which otherwise would be marginalised or excluded. Twitter is a helpful tool to investigate the hikikomori phenomenon since those affected often use social media as an online ‘refuge’ [1, 25]. Previous studies of hikikomori using infodemiology explored contents and perceptions related to hikikomori on Twitter in Japanese and some Western languages, finding personal stories as the most posted content, and several mentions of hikikomori in non-Japanese Western languages [7, 8]. Understanding the content of tweets related to the hikikomori phenomenon provides valuable information on how this social issue is perceived and discussed among different people.

Although hikikomori was considered a typical phenomenon related to Japanese culture [2, 26, 27], over the years different studies have been conducted to investigate this phenomenon in other countries [1, 7, 28, 29]. While Portuguese is the sixth most spoken language in the world, to this day, no studies have analysed this phenomenon in this language.

The main aim of this study has been to explore contents related to the hikikomori phenomenon in the Portuguese language on Twitter. In particular, this study addressed the following research questions: (1) How do Twitter users express their views on hikikomori and what emotions do they associate with it? (2) Has the frequency of tweets about the hikikomori phenomenon changed over time, including during the COVID-19 pandemic? (3) What type of content related to hikikomori generates more interest on Twitter? (4) Are there any geographical differences in Portuguese-speaking countries regarding the hikikomori phenomenon tweets?

## Methods

### Research strategy

The research strategy focused on the collection and content analysis of Portuguese-language tweets about hikikomori. We included tweets that met the following criteria: (1) public (not private/protected) tweets; (2) use the keyword *hikikomori*; (3) tweets posted between 1st January 2008, and 19th October 2022; and (4) text in Portuguese language. The exclusion criteria were: (1) the majority of the text in the tweet in a language other than Portuguese; (2) tweets with only a link or image without any text. The tool used for collecting tweets was Tweet Binder, which has been widely used in previous research and provides access to 100% of public tweets [30, 31]. This tool provides the tweet text, count of retweets and likes for each tweet, as well as the date of publication, a link to the tweet in its context, user description, and geolocation data (obtained from the biography [bio] of the account that published the tweet). Regarding geolocation, we have gathered data at the country level. To enable comparison of the number of tweets posted in each country, we have grouped them into five continents: Americas (North, Central and South America), Europe, Africa, Asia and Oceania. The number of retweets and likes generated by each tweet were analysed as an indicator of user interest in a given topic.

### Content analysis process

All tweets in our database were analysed using a content analysis procedure that consisted of creating codes and categories. We created a codebook to characterise each tweet, where each tweet was analysed according to the categories including: i) whether content was from a positive (tweets that express solidarity, self-disclosure, encouragement, gratitude, enthusiasm and pride) or negative perspective (tweets that express blame, stigma, and negative opinions); ii) whether it was about information relating to the hikikomori phenomenon; iii) whether it was an account of personal stories, or whether it was about curiosities of the phenomenon. Table 1 provides a detailed characterisation of the categories and examples of tweets that fall into these categories. The Twitter users were classified into three users types: “individuals with hikikomori” (people who describe themselves as hikikomori), “family and friends” (including family members and close friends or acquaintances), and “others” (unspecified as not fitting the previous types).

**Table 1:** Detailed characterisation of the categories and examples of tweets that fall into these categories

CODE	DEFINITION	EXAMPLES OF TWEETS IN PORTUGUESE	TRANSLATION OF TWEETS IN ENGLISH
Concept information	Tweets that attempt to inform about what the hikikomori phenomenon is	“Você sabe o que é #hikikomori ? Hikikomori é um fenômeno social recente na história do Japão. As autoridades do país definiram que pessoas em reclusão social a mais de seis meses são portadores da doença.”	"Do you know what #hikikomori is? Hikikomori is a recent social phenomenon in Japanese history. The country's authorities have defined people in social withdrawal for more than six months as having the disease."
Online Support	Tweets offering online support to people with hikikomori	“Participe do grupo Hikikomori Brasil no Facebook. #Hikikomori #Otaku”	"Join the Hikikomori Brazil group on Facebook. #Hikikomori #Otaku"
Personal stories	Tweets about your day to day life as a hikikomori	“Por hoje é só a cadelinha aqui precisa dormir por que acorda cedo, vou tentar atualizar amanhã mas não prometo nada se não é só sábado mesmo. Obrigado por lerem meus dengo e Views em #HIKIKOMORI#”	"That's it for today, the little dog here needs to sleep because she wakes up early, I'll try to update tomorrow but I can't promise anything if it's not just Saturday. Thanks for reading my dengo and Views on #HIKIKOMORI#"
Curiosities	Tweets reporting curiosities about the phenomenon	“[…] lançou a música “#HIKIKOMORI”! O nome da música é um termo japonês que retrata pessoas que se isolam da sociedade evitando contato com as pessoas […]” “#HIKIKOMORI: pessoa reclusa, isolada de todos e tudo. o JAPÃO sabia que tinha 541 mil deles entre 15 e 39 anos. uma pesquisa acaba de revelar que há 613 mil entre 40 e 64 anos. um caso especial são as “casas 8050”, pais de 80 + cuindando de reclusos de 50 + .”	"[…] has released the song "#HIKIKOMORI"! The name of the song is a Japanese term for people who isolate themselves from society by avoiding contact with people […]” "#HIKIKOMORI: a reclusive person, isolated from everyone and everything. JAPAN knew that there were 541,000 of them between the ages of 15 and 39. a survey has just revealed that there are 613,000 between the ages of 40 and 64. a special case is the "8050 houses", 80 + parents looking after 50 + inmates."

### Application of artificial intelligence to evaluate tweets

Recent technological advancements have led to the development of artificial intelligence (AI), including machine learning (ML) [32] and deep learning (DL) [33]. Neural networks, inspired by human brain neurons, are extensively used in various applications, such as natural language processing (NLP) [34], weather prediction [35], coronavirus detection [36], and image object detection [37]. In this study, a pre-trained neural network called BERTWEET [38], trained on 850 million English tweets, was employed to classify hikikomori-related tweets into different categories.

Before applying the BERTWEET network, the tweet database underwent preprocessing steps. Non-English tweets were translated into English using Google Translator since the network is trained only on English tweets. Previous studies have demonstrated that employing Google Translator to translate text into English and subsequently using a model trained on English tweets can enhance the performance of machine learning models [39–41]. The tweets were then normalised by removing special characters, separating negative tenses, and eliminating repeated characters. As BERTWEET was not originally trained to classify the desired categories, a process called fine-tuning was conducted. The manually classified tweets were randomly split into training and testing subsets, with 75% used for fine-tuning the network and 25% for validation. We validated the correct performance of the model in the validation set by computing the weighted F1 score, consistently achieving a score above 0.8 across all categories. This methodology, previously employed with positive outcomes [42], was employed to ensure the fine-tuned BERTWEET model performed well on the database. The fine-tuned BERTWEET model was then used to categorise the remaining tweets that had not been manually classified.

Additionally, the emotions expressed in the tweets were analysed using a pre-trained neural network called emotion-english-distilroberta-base [43]. This network, capable of detecting Ekman's six basic emotions plus neutral, was applied to the translated and normalised dataset of 13,915 tweets [44]. The emotion-english-distilroberta-base model, previously used in other research studies, does not require additional fine-tuning as it serves the same purpose for which it was originally trained [45, 46]. The number of likes and retweets each tweet generated, the date and time of each tweet, a permanent link to the tweet, and a description of each user's profile were collected. The nature of users who posted tweets was determined according to the available information (tweet content, description of the user's profile, or Twitter identifier).

### Statistical analysis

Descriptive statistics were used to summarise tweets, likes, and retweets of users related to the content topics, tone, user type, emotions, and location. Correlation coefficients were determined to measure the strength and direction of their association. We also investigated the number of tweets and retweets generated by Twitter users by the days of the week grouped by user type. Additionally, we conducted an emotional analysis to graphically represent Twitter users' emotions by the type of user; time trends were used to describe the number of all tweets posted by continents between 2008–2022 as well as to illustrate the number of tweets posted before and after the COVID-19 pandemic; lastly, the proportions of Tweets posted by type of users across continents were graphically represented. All analyses were performed with STATA version 15 (StataCorp LP).

### Ethical considerations

This study used publicly available tweets. This study received approval from the Ethics Committee of the Biomedical Sciences Institute Abel Salazar at the University of Porto (Ref 2023/CE/P03/(P401/CETI/ICBAS)).

## Results

### Overall Tweets, Likes, and Retweets

Among a total of 13,915 tweets posted in Portuguese about hikikomori, in terms of tone 3,184 (22.9%) were classified as “positive”, and 10,731 (77.1%) as “negative”. The content of the tweets was classified as “curiosities” (*n* = 9,818, 70.6%), “personal stories” (*n* = 3,675, 26.4%) or “concept information” (*n* = 422, 3.0%) (Table 2).

**Table 2:** Number of tweets, retweets, and likes related to the content topics, tone, user type, emotions, and location

	**Tweets**		**Likes**		**Retweets**	
	**n**	**%**	**n**	**Number Likes/Number Tweets**	**n**	**Number Retweets/Number Tweets**
**Overall**	13,915	100	21,307	–	5,359	–
***User type***						
**Individuals with hikikomori**	5,571	40.0	3,953	0.71	404	0.07
**Family and friends**	4,653	33.4	5,013	1.08	346	0.07
**Others**	3,691	26.5	12,341	3.34	4,609	1.25
***Content***						
**Concept Information**	422	3.0	1,694	4.01	322	0.76
**Personal Stories**	3,675	26.4	2,862	0.78	459	0.12
**Curiosities**	9,818	70.6	16,751	1.71	4,578	0.47
***Tone***						
**Positive**	3,184	22.9	3,735	1.17	1,662	0.53
**Negative**	10,731	77.1	17,572	1.64	3,697	0.34
***Emotions***						
**Anger**	1,114	8.0	1,302	1.17	222	0.2
**Disgust**	273	2.0	2,695	9.87	1,012	3.71
**Fear**	1,319	9.5	2,734	2.07	941	0.71
**Joy**	2,453	17.6	3,039	1.24	1,413	0.58
**Neutral**	4,991	35.9	6,706	1.34	770	0.15
**Sadness**	1,822	13.1	2,498	1.37	751	0.41
**Surprise**	1,943	14.0	2,333	1.20	250	0.13
***Continents***						
**Americas**	487	3.5	226	0.46	140	0.29
**Europe**	553	4.0	2,892	5.23	1,098	1.99
**Africa**	91	0.7	55	0.6	13	0.14
**Asia**	377	2.7	426	1.13	141	0.37
**Oceania**	7	0.1	20	2.86	2	0.29
**Missing**	12,400	89.1	17,688	1.4	3,965	0.32

Within the user type, these tweets were classified as “individuals with hikikomori” (*n* = 5,571, 40.0%), “family and friends” (*n* = 4,653, 33.4%), or “others” (*n* = 3,691, 26.5%).

In regard to the emotion analysis, the higher proportion of tweets were “neutral” (*n* = 4,991, 35.9%). The emotion “joy” was present in 2,453 (17.6%) tweets, “surprise” in 1,943 (14.0%) tweets, “sadness” in 1,822 (13.1%) tweets, “fear” in 1,319 (9.5%) tweets, “anger” in 1,114 (8.0%) tweets, and “disgust” in 273 (2.0%) tweets.

Regarding the world regions, the majority of tweets (*n* = 12,400; 89.1%) did not report their location (“Missing”). Among the tweets with available location, the greater proportion of tweets was posted in “Europe” (*n* = 553, 4.0%). From the rest of the tweets, 487 (3.5%) tweets were posted in the “Americas”, 377 (2.7%) tweets were posted in “Asia”, 91 (0.7%) tweets were posted in “Africa” and 7 (0.1%) tweets were posted in “Oceania”.

There was a total of 21,307 likes and 5,359 retweets of hikikomori-related phenomenon in Portuguese. Regarding content, “curiosities” showed the highest proportion of the number of likes and retweets. Tweets posted in a positive way have more likes and retweets than tweets posted in a negative way. The "others" type of user presented a higher proportion of number of likes and retweets and, and “disgust” feelings was the sentiment with the most likes and retweets. “Europe” was the continent with the highest number of likes and retweets of hikikomori-related tweets in Portuguese-language.

The full details of the frequency of these tweets are reported in Table 2.

### Content topics

The information regarding content topics and tweets is illustrated in Table 3, categorised by user type, emotions, continents and tone.

**Table 3:** Content topics related to tweets classified according to the type of user, emotions, continents, and tone

	**Tweets related to Content**					
	**Concept information**		**Personal stories**		**Curiosities**	
	**n**	**%**	**n**	**%**	**n**	**%**
**Overall**	422	3.0	3,675	26.4	9,818	70.6
***User type***						
**Individuals with hikikomori**	9	2.1	3,536	96.2	2,026	20.6
**Family and friends**	15	3.6	112	3.1	4,526	46.1
**Others**	398	94.3	27	0.7	3,266	33.3
***Tone***						
**Positive**	25	5.9	1,010	27.5	2,149	21.9
**Negative**	397	94.1	2,665	72.5	7,669	78.1
***Emotions***						
**Anger**	22	5.2	258	7.0	834	8.5
**Disgust**	4	1.0	94	2.6	175	1.8
**Fear**	75	17.8	391	10.6	853	8.7
**Joy**	27	6.4	519	14.1	1,907	19.4
**Neutral**	204	48.3	1,058	28.8	3,729	38.0
**Sadness**	53	12.6	876	23.8	893	9.1
**Surprise**	37	8.8	479	13.0	1,427	14.5
***Continents***						
**Americas**	47	11.1	106	2.9	334	3.4
**Europe**	17	4.0	137	3.7	399	4.1
**Africa**	7	1.7	22	0.6	62	0.6
**Asia**	21	5.0	107	2.9	249	2.5
**Oceania**	0	0	4	0.1	3	0
**Missing**	330	78.2	3,299	89.8	8,771	89.3

*Concept information*: In terms of tone, 397 (94.1%) tweets were classified as “negative”, and 25 (5.9%) as “positive”. Within the user type, these were made by “others” in 398 (94.3%), “family and friends” in 15 (3.6%) tweets and “individuals with hikikomori” in 9 (2.1%) tweets. With respect to emotion analysis, the majority of tweets were “neutral” (*n* = 204, 48.3%). The emotion “fear” was present in 75 (17.8%) tweets, “sadness” in 53 (12.6%) tweets, “surprise” in 37 (8.8%) tweets, “joy” in 27 (6.4%) tweets, “anger” in 22 (5.2%) tweets and “disgust” in 4 (1.0%) tweets. Across the world, a total of 330 (78.2%) tweets did not report their location (“Missing”), 47 (11.1%) tweets were posted in “Americas”, 21 (5.0%) tweets were posted in “Asia”, 17 (4.0%) tweets were posted in “Europe”, 7 (1.7%) tweets were posted in “Africa”, and 0 (0%) tweets were posted in “Oceania”.

*Personal stories*: In terms of tone, 2,665 (72.5%) tweets were classified as “negative”, and 1,010 (27.5%) tweets as “positive”. Within the user type, these were made by “individuals with hikikomori” in 3,536 (96.2%) tweets, “family and friends” in 112 (3.1%), and “others” in 27 (0.7%) tweets. With respect to emotion analysis, most tweets were “neutral” (*n* = 1,058, 28.8%). The emotion “sadness” was present in 876 (23.8%) tweets, “joy” in 519 (14.1%) tweets, “surprise” in 479 (13.0%) tweets, “fear” in 391 (10.6%) tweets, “anger” in 258 (7.0%) tweets and “disgust” in 94 (2.6%) tweets. Worldwide, of 3,299 (89.8%) tweets we have no report of their location (“Missing”), 137 (3.7%) tweets were posted in “Europe”, 107 (2.9%) tweets were posted in “Asia”, 106 (2.9%) tweets were posted in “Americas”, 22 (0.6%) tweets were posted in “Africa” and 4 (0.1%) tweets were posted in “Oceania”.

*Curiosities*: In terms of tone, 7,669 (78.1%) tweets were classified as “negative”, and 2,149 (21.9%) as “positive”. Within the user type, these were made by “family and friends” in 4,526 (46.1%), “individuals with hikikomori” in 2,026 (20.6%) tweets, and “others” in 3,266 (33.3%) tweets. With respect to emotion analysis, the majority of tweets were “neutral” (n = 3,729, 38.0%). The emotion “joy” was present in 1,907 (19.4%) tweets, “surprise” in 1,427 (14.5%) tweets, “sadness” in 893 (9.1%) tweets, “fear” in 853 (8.7%) tweets, “anger” in 834 (8.5%) tweets and “disgust” in 175 (1.8%) tweets. Worldwide, we had no report of the location of 8,771 (89.3%) tweets (“Missing”), 399 (4.1%) tweets were posted in “Europe”, 334 (3.4%) tweets were posted in “Americas”, 249 (2.5%) tweets were posted in “Asia”, 62 (0.6%) tweets were posted in “Africa” and 3 (0%) tweets were posted in “Oceania”.

### Emotion analysis

**Fig. 1:**
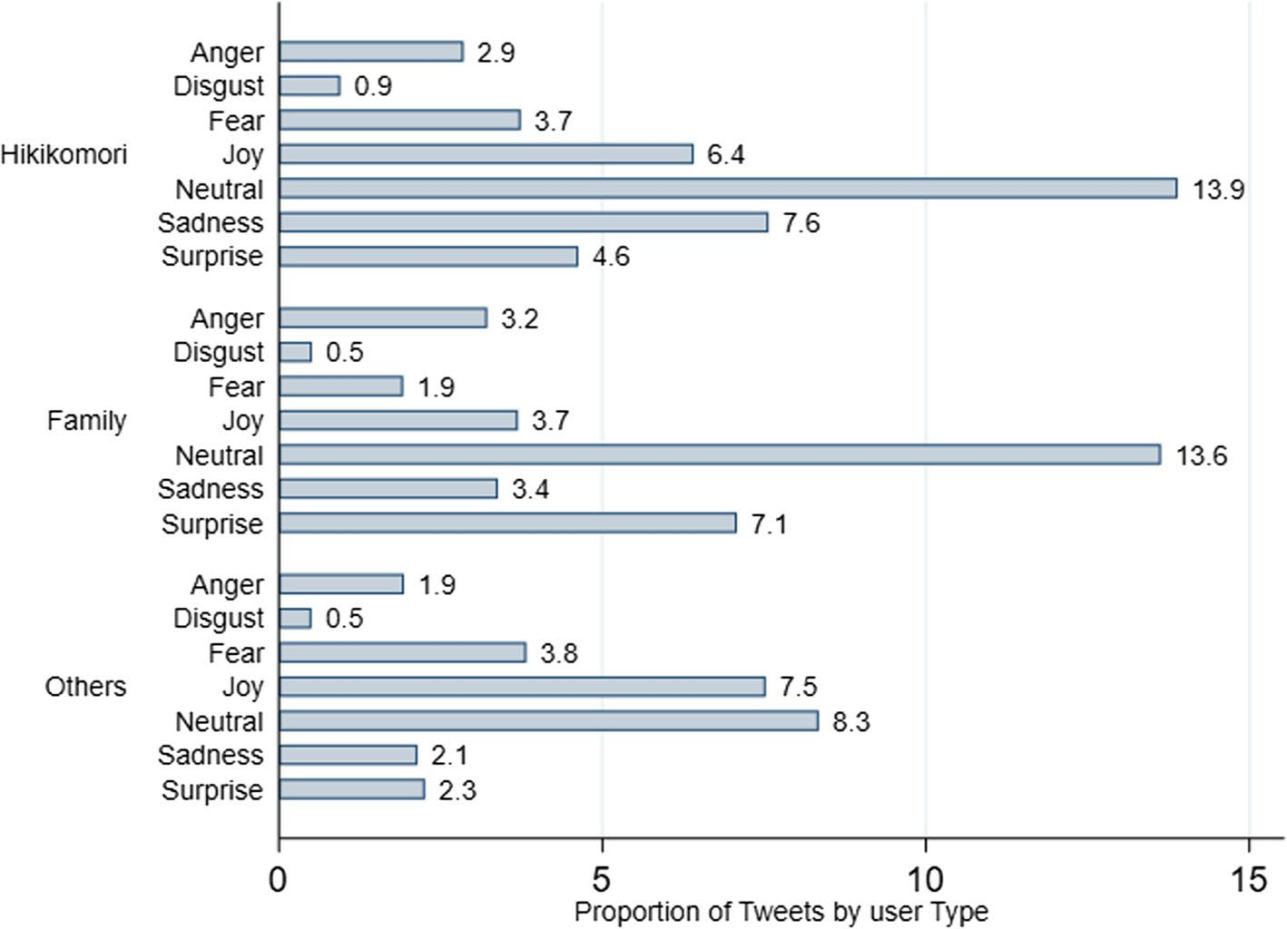
Proportion of type of user by considered emotions of tweets

Figure 1 displays the Twitter users' emotions by type of user and reports that in all user types, the majority of tweets were “neutral”, whereas “disgust” emotion was the least frequent. Among the “individuals with hikikomori” users, the second most frequent emotion was “sadness”, whilst in the “family and friends” the second most frequent emotion was “surprise”, and in “others” types of users the second most prevalent emotion was “joy”.

### Number of tweets over time (2008–2022)

**Fig. 2:**
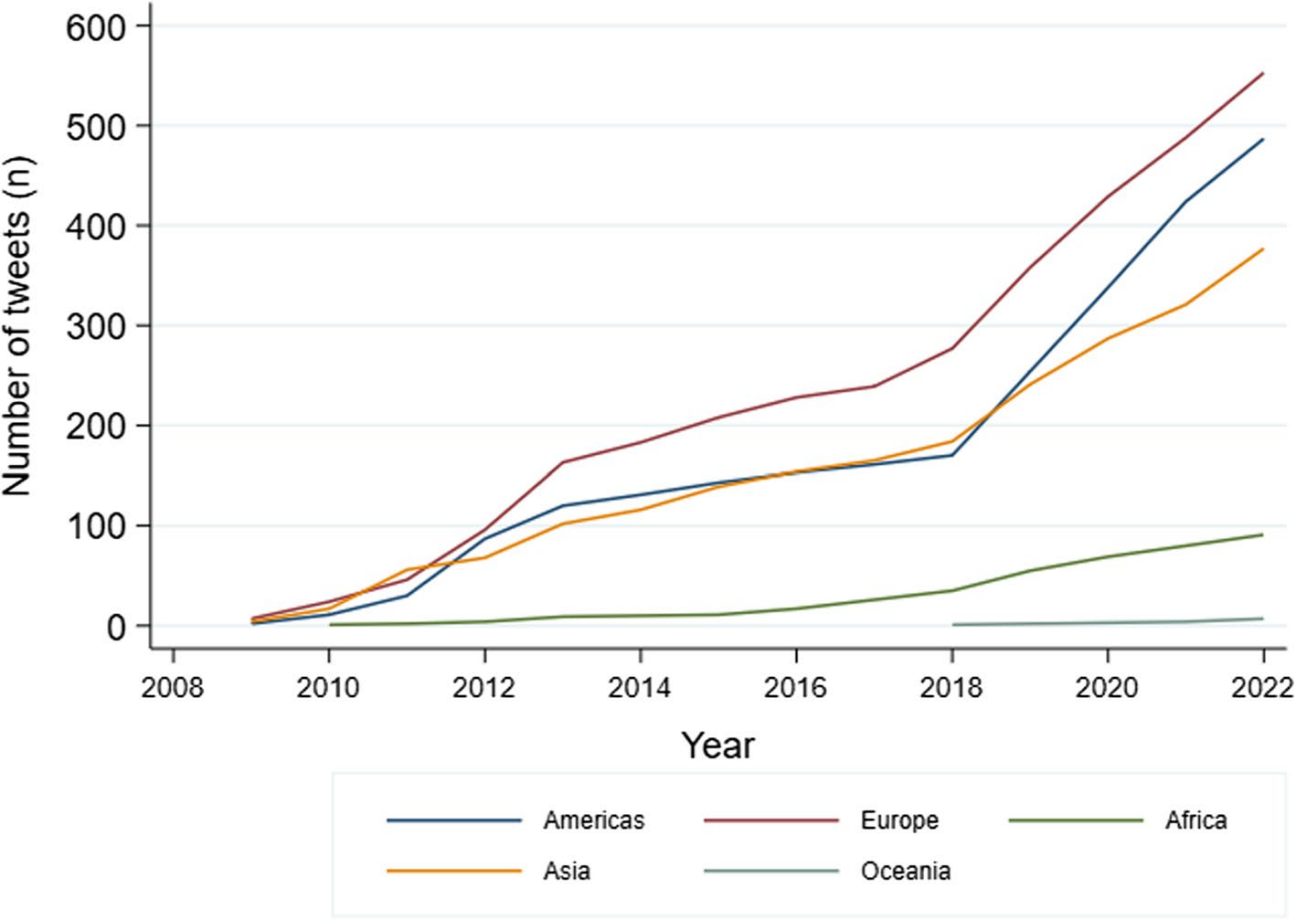
Temporal evolution of tweets published from 2008 to 2022 by continents

Over the years (Fig. 2), there was a progressive increase in the number of tweets about hikikomori in Portuguese on all continents except in Oceania, where tweets were only retrieved since 2018 and where there are fewer tweets. Particularly in the Americas, Europe, and Asia, there was a higher increase in the number of tweets as the years passed.

**Fig. 3:**
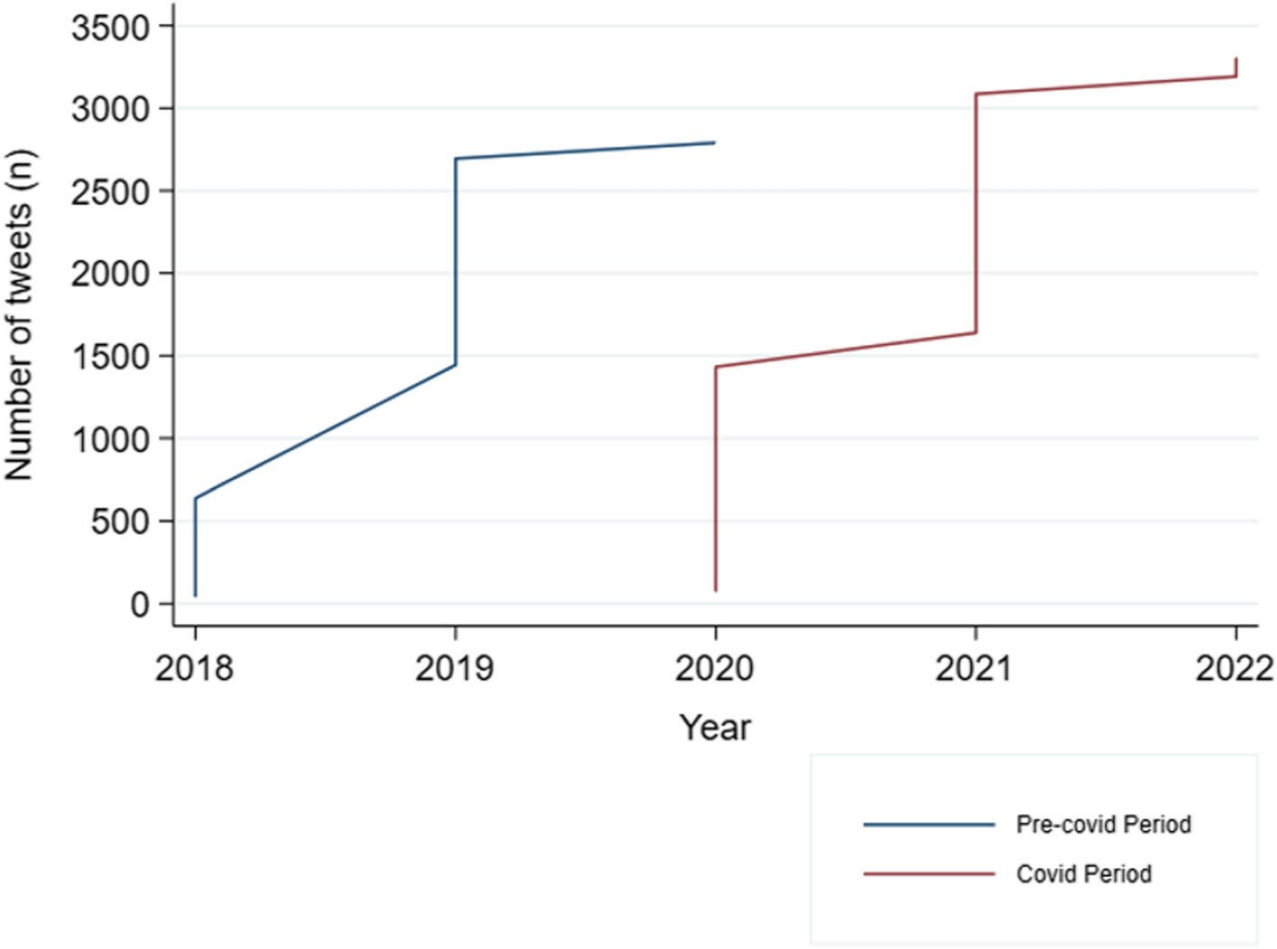
Time trend of all tweets posted 2 years before and 2 years after the beginning of the COVID-19 pandemic (1st March 2020). In this figure, the line represents the frequency of tweets posted by users

Regarding the COVID-19 pandemic period, a difference in the number of tweets posted before and after the pandemic can graphically be observed. During and after the pandemic the number of tweets was higher (Fig. 3).

### Continents

**Fig. 4:**
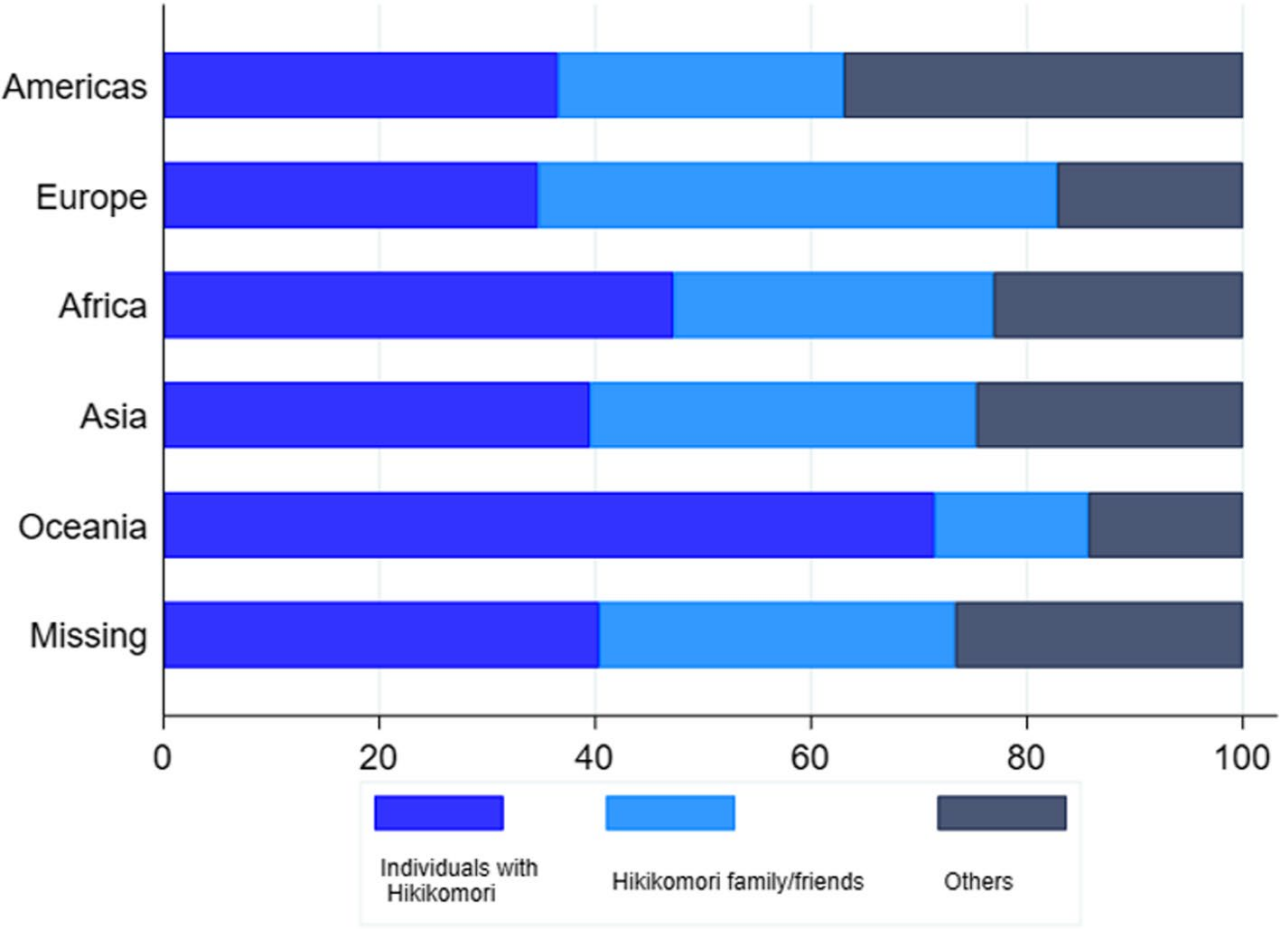
Proportion of tweets published by each user type (Y-axis) according to the continent (X-axis)

In Africa, Asia and Oceania, "individuals with hikikomori" were the type of user posting the most tweets. In America, there was an equal distribution of tweets posted by “individuals with hikikomori” and "others". In Europe, the majority of tweets were posted by “family and friends” (Fig. 4).

## Discussion

### Key findings

The number of likes and retweets retrieved reflects an interest in the hikikomori phenomenon in Portuguese-speaking Twitter users. Regarding the content, tweets about curiosities were the most frequent, with the majority reporting hikikomori as something negative. Concerning the user type, “individuals with hikikomori” posted more tweets about the story of their day (personal stories), while for “family and friends” of people with hikikomori, curiosities was the topic that got more attention, and “others” posted more tweets about the concept of hikikomori itself. Among all the types of users, “individuals with hikikomori” were the most frequent. In terms of emotions, most tweets were neutral, with the least frequent emotion being disgust.

The knowledge of the hikikomori phenomenon in Portuguese language is globally spread, with users in Europe posting the most tweets and users in Oceania the least. The distribution of the different user types was relatively homogeneous, with most continents having more tweets posted by “individuals with hikikomori”. Many people seemed to join social networks to occupy their time during the COVID-19 period [15], increasing the number of tweets posted during the pandemic compared to before.

### Comparison with the other literature

A study conducted with psychiatrists from several countries, supported the notion that the hikikomori phenomenon is global [27, 47]. Consistent with our findings, a previous study [7] identified Twitter contents suggesting the existence of hikikomori in Western countries based on the languages analysed (Catalan, English, French, Italian, and Spanish). The number of tweets posted from different continents has increased significantly over the years. This phenomenon may be explained, on the one hand, as people are using Twitter more to express their thoughts and feelings, and on the other hand, because the hikikomori phenomenon is becoming worldwide a more known and trending topic [1, 25, 47]. A study exploring the general population’s attitudes toward prison volunteering, demonstrated that users posted more tweets in Portuguese about volunteering in prisons in America (Ferrão Nunes et al.: Public discourse towards volunteering in prisons: An infodemiology study of Twitter data, submitted), whereas our study revealed that users from Europe posted the most. This may be related to the fact that people from Europe are more familiar with the term hikikomori than people from other continents.

While a previous study [8] of tweets about hikikomori in Japanese showed that personal stories were the most posted content, in our study, curiosities about hikikomori were predominant, suggesting that the hikikomori phenomenon has generated more interest among people since the users who posted the tweets know some curiosities about this phenomenon.

The interest of users in a specific topic can be assessed by analysing the number of retweets and likes that each tweet generates [48]. A study [8] that explored this phenomenon by analysing tweets had more likes than retweets, similar to our study. This could be attributed to the fact that people are more comfortable to show their interest in the subject, but less inclined to actively share the tweets for wider dissemination.

Two previous studies [7, 8] have additionally shown that the majority of tweets have reported hikikomori as a problem, which corroborates in the findings of our study, suggesting widespread awareness of the seriousness of the phenomenon.

### Implication of the findings for future policies and research

Twitter is a helpful tool to study the hikikomori phenomenon since the affected individuals often use social networks as a refuge [1, 25]. Internet platforms allow us to reach socially withdrawn youth, and in this way, warn of risks and provide support.

This study suggests, similarly to other studies, that the hikikomori phenomenon is not only restricted to Japan [7, 8, 27, 47]; it specifically shows that the phenomenon is also known and spoken about among Portuguese speakers all over the world.

This study provides valuable insights into the hikikomori phenomenon on Twitter in Portuguese, offering implications for future research and policies aimed at addressing this complex social issue. These findings are a call to action to further investigate and develop methods to assist individuals with hikikomori who have been identified through the Twitter platform. The identification of individuals with hikikomori tendencies in this study underscores the need to provide them with the necessary support and resources to help these individuals seek help. Future research could focus on effective intervention and support strategies tailored to the needs of individuals affected by hikikomori. Exploring the potential of social media platforms like Twitter to provide psychosocial support and establish a good support network for individuals affected by hikikomori, as well as their families and friends, encouraging professional help as well, is another area for future investigation. Understanding how these platforms can be utilised to promote mental health and well-being in the context of hikikomori is crucial for developing effective support systems. It is also essential to promote education on the subject, alerting the public to its risks and consequences, and raising awareness of ways to prevent its development.

Future studies could consider conducting comparative analyses by examining data from Twitter and other social media platforms in other languages and countries where the hikikomori phenomenon has not yet been researched. This approach would provide a more comprehensive understanding of the global impact of hikikomori and its manifestations in different cultural contexts.

### Strengths and limitations

This is the first study to investigate tweets related to hikikomori in the Portuguese language and to investigate its geographical differences. However, this study has some limitations. First, using a specific keyword may have limited our collection of tweets, since users may have used another term to refer to the hikikomori phenomenon, and thus tweets that refer to it in a different way may not have been collected. Furthermore, the potential deletion of tweets over time, whether by the social network, users, or through account deletion or protection, may have resulted in the exclusion of valuable data from the analysis, potentially impacting the comprehensiveness of the study findings. Additionally, the increase in tweets observed after the pandemic may not be related to users' interest in the topic. Therefore, it may not be conclusive that the pandemic has significantly impacted the level of interest in the hikikomori phenomenon compared to other topics. Another limitation of the study is that a significant portion of the tweets lacked geographical location data, limiting the ability to gain insights into regional variations of the hikikomori phenomenon among Portuguese speakers. Finally, whilst we analysed the number of retweets and likes generated by each tweet as an indicator of user interest in a given topic, these engagement metrics can be influenced by other factors and thus may not truly match user interest.

## Conclusions

These findings demonstrate that the hikikomori phenomenon is actively discussed and appears to be prevalent among Portuguese speakers on all continents of the world. This supports and enhances our understanding of the globalisation of this phenomenon. The majority of tweets seemed to originate from individuals apparently affected by hikikomori. They reported curiosities about the phenomenon and discussed it in a negative light, suggesting that most people perceive it as a problem that needs to be addressed. Tweets about hikikomori in the Americas, Asia, and Europe have been steadily increasing in recent years, without a noticeable surge during the COVID-19 pandemic. This suggests that the prevalence of hikikomori is gradually growing in these continents. This study underscores the need to provide individuals with hikikomori tendencies with the necessary support and resources to improve their quality of life. It also offers implications for future research and policies addressing this complex social issue on Twitter.

## Data Availability

The datasets analysed available from the corresponding author upon reasonable request.

## Glossary

Hikikomori: Term used to describe the social phenomenon of individuals, often young adults, who withdraw from social life and isolate themselves at home for a prolonged period, avoiding social interaction.
Infodemiology: Refers to the study of how information spreads and impacts public health, especially on the internet. Analyses patterns of information disclosure to understand how it affects people's knowledge and behaviour.
Machine Learning: Subset of artificial intelligence that involves the development of algorithms and statistical models that enable computers to perform tasks without explicit programming. It relies on patterns and inference from data to improve performance over time.
Deep Learning: A specialised area of machine learning that involves using neural networks with multiple layers (deep neural networks) to model and solve complex problems. It is particularly effective in tasks such as image and speech recognition.
BERTweet: Natural language processing model based on BERT (Bidirectional Encoder Representations from Transformers) architecture, specifically tailored for processing and understanding Twitter data. It enhances language understanding in a social media context.
Emotion-English-Distilroberta-Base: Pre-trained language model based on the Distilroberta architecture, specialised in recognizing and understanding emotions in English text. It is trained to analyse and categorise emotions expressed in written content.
